# An Unusual Presentation of Chondrosarcoma of the Clavicle With Horner's Syndrome

**DOI:** 10.1080/13577140410001710530

**Published:** 2004

**Authors:** V. Kapoor, V. Sudheer, M. Waseem, A. S. Cole, D. F. Weeden

**Affiliations:** Department of Trauma and Orthopaedics Department of Thoracic Surgery Southampton University Hospital Tremona Road, Shirley Southampton SO16 6YD UK


*Sarcoma*, June/September 2004, *VOL. 8, NO. 2/3, 87–89*

Taylor & Francis healthsciences

CASE REPORT

### An unusual presentation of chondrosarcoma of the clavicle with Horner's syndrome

V. KAPOOR, V. SUDHEER, M. WASEEM, A. S. COLE & D. F. WEEDEN

*Department of Trauma and Orthopaedics, Department of Thoracic Surgery, Southampton University Hospital, Tremona Road, Shirley, Southampton SO16 6YD, UK*

### Introduction

We report a unique case of symptomatic chondrosarcoma of the medial end of the clavicle resulting in pain and Horner's syndrome. To our knowledge this is the first reported case in the literature of Horner's syndrome secondary to a clavicular tumour.

Osteochondroma, a frequent benign tumour of the bone, is in many cases asymptomatic. The reported risk of malignant transformation in a solitary osteochondroma is approximately 1%.[1] Localisation in the clavicle is extremely rare. The lesion in this case was primarily an osteochondroma that underwent malignant transformation becoming symptomatic.

### Case report

A 28-year-old soldier presented with a 3-year history of left-sided shoulder pain and ptosis. The pain was related to physical activity. There was no rest or night pain. There was no history of trauma to the shoulder.

Clinical examination revealed tenderness at the medial end of clavicle, swelling in the supraclavicular fossa and an associated Horner's syndrome. Radiographs showed a soft tissue mass in the apical region. A CT scan with three-dimensional reconstruction (Figs. 1 and 2) confirmed a bony lesion arising from the posterior aspect of the medial end of the clavicle.

The growth was excised through an anterior approach. The lesion was noted to be compressing the neurovascular structures in the root of the neck but not directly involving them. The lesion was lobulated and well encapsulated with a large cartilaginous cap (10 mm) typical of a large osteochondroma measuring $50 \times 45 \times 25$ mm. The lesion was completely excised and confirmed by subsequent histology.

Histological examination revealed a low grade (I) chondrosarcoma arising from an osteochondroma. On the advice of the Oncologists no further treatment was undertaken.

Follow-up at 1 year revealed no recurrence and the patient was pain free returning to full time work and competitive rowing. However, his ptosis has persisted.

### Discussion

Primary bone tumours and tumour-like lesions of the clavicle are uncommon. A review by Klein *et al.*[2] found that only 0.45% of more than 13 000 primary bone tumours involved the clavicle. Smith *et al.*[3] in a review of 35 primary bone tumors of the clavicle that have been treated at the Memorial Sloan-Kettering Cancer center, reported only five benign lesions. Primary bone tumours of the clavicle are more likely to be malignant than benign.

This described case is unique as it caused Horner's syndrome (oculosympathetic palsy). The lesion involved the second order neuron (preganglionic) leading to disruption in the sympathetic supply. The second order neuron passes from the ciliospinal center of Budge to the superior cervical ganglion in neck. During its long course, it is closely related to the apical pleura where it may be damaged by apical lesions in the lung, neck swellings or surgery.[4] It is therefore essential to examine the supraclavicular fossa for such swellings in patients presenting with Horner's syndrome. In this case the lesion was closely related to the apical pleura thereby compressing the second order neuron.

Correspondence to: Mr. V. Kapoor, Orthopaedic SpR, C/o Mr. Harley's secretary, 'F' level MP 45, Orthopaedics Department, Southampton General Hospital, Tremona Road, Shirley, Southampton SO16 6YD, UK. Tel: +44-2380-777222, Ext. 6795; Fax: +44-2380-794414; E-mail: Vkaboor@aol.com

1357-714X print/1369–1643 online © 2004 Taylor & Francis Ltd
DOI: 10.1080/13577140410001710530

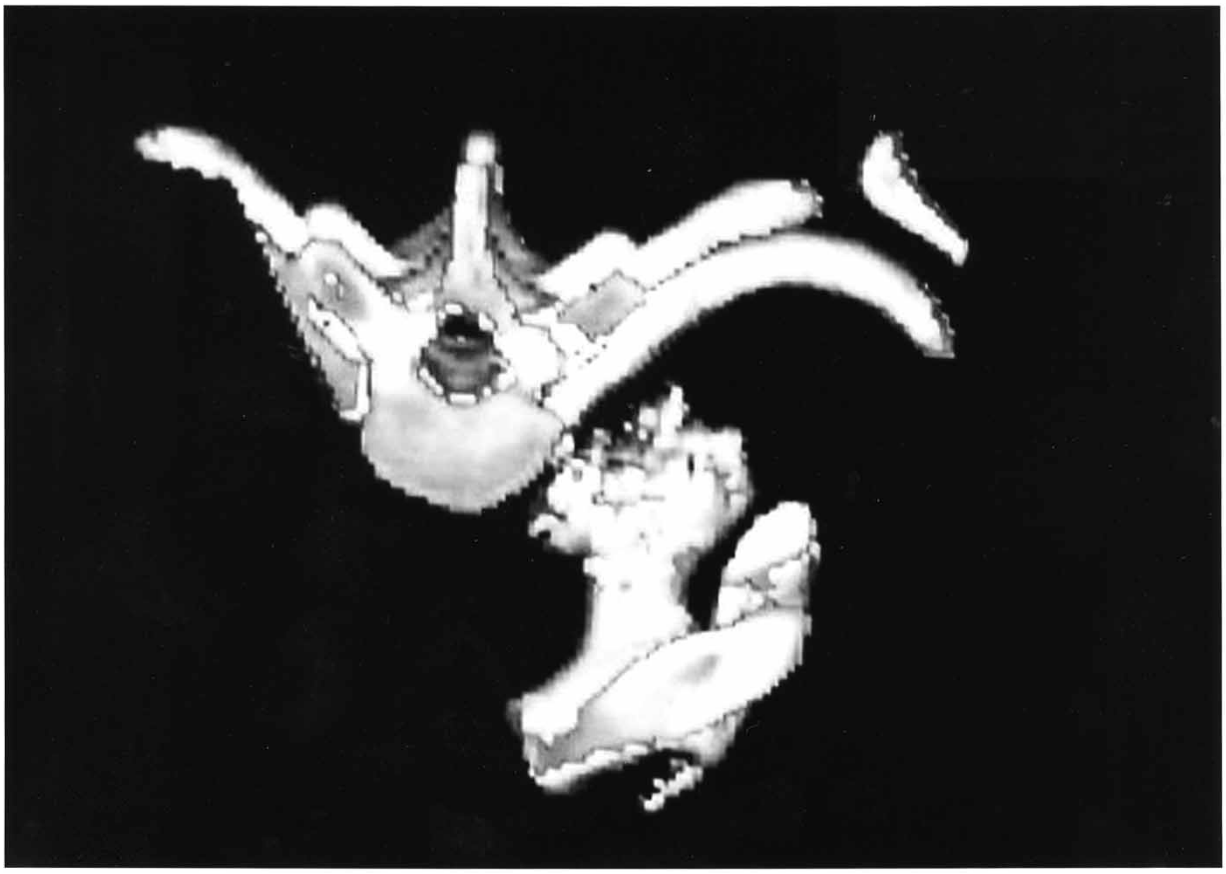

**Fig. 1.** *Selected CT scan image showing a pedunculated chondrosarcoma arising from the medial end of the clavicle, with an irregular cap.*

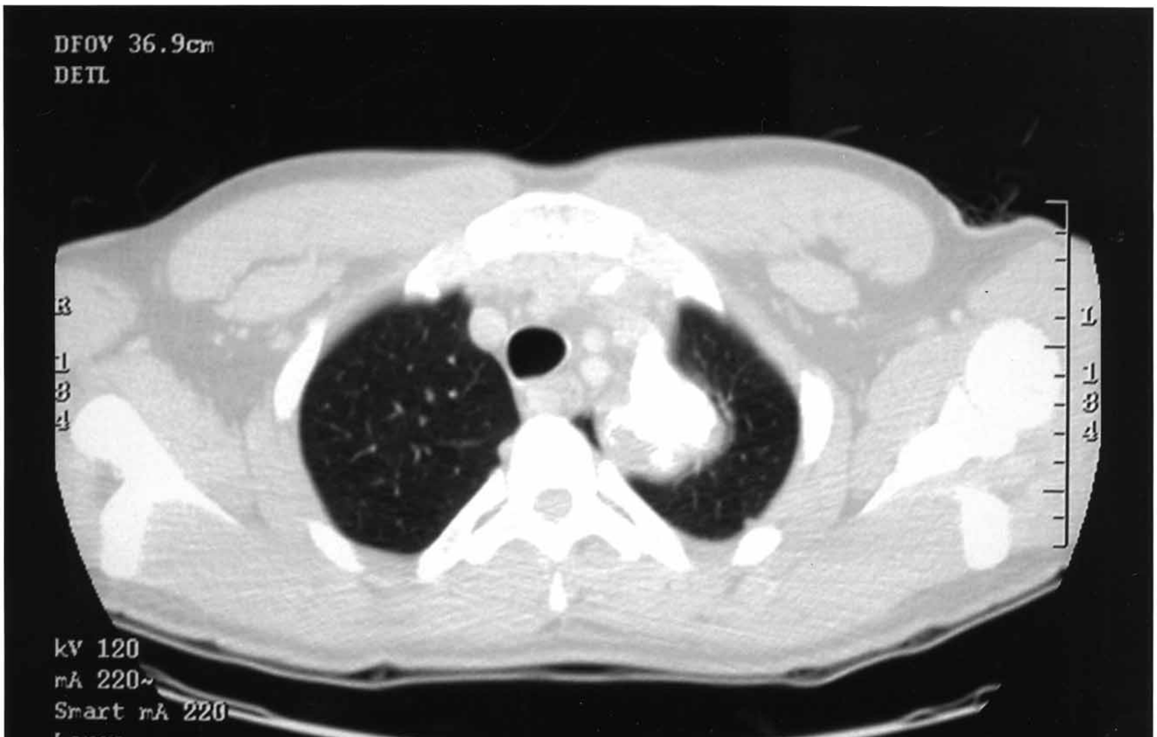

**Fig. 2.** *Contrast-enhanced axial image of the lesion on thoracic CT scan.*

Solitary osteochondroma, a frequent benign tumour of bone is only very rarely located in the clavicle. An osteochondroma may become symptomatic as a result of a fracture of the stalk, an inflammatory reaction of the overlying bursa, impingement against soft tissue, compression of peripheral nerves or the spinal cord and malignant transformation.[5] Removal is indicated if the tumour is unsightly, is producing pain and disability, shows an abnormal increase in size or has roentgenographic features suggestive of malignancy.

In this case the long-standing osteochondroma had undergone malignant change, became symptomatic and presented with shoulder pain and ptosis. It highlights the need to examine the supraclavicular fossa in patients presenting with Horner's symdrome.

### References

1. Unni KK. *Dahlin's Bone tumor*. New York: Lippincott-Raven, 1996.
2. Klein MJ, Luskin R, Becker MH, Antopol SC. Osteoid osteoma of the clavicle. *Clin Orthop* 1979; 143: 162–4.
3. Smith J, Yuppa F, Watson RC. Primary tumors and tumor-like lesions of the clavicle. *Skeletal Radiol* 1988; 17(4): 235–46.
4. Kanski JJ. *Clinical Ophthalmology*. New York: Butterworth Heinemann, 1999.
5. Ogawa K, Yoshida A, Michimasa UI. Symtomatic osteochondroma of the clavicle. *J Bone Jt Sorg* 1999; 61A(3): 404–8.
